# Editorial: Natural products and nanotechnology: next-generation therapies for infectious diseases

**DOI:** 10.3389/fphar.2025.1682331

**Published:** 2025-09-01

**Authors:** Dolores R. Serrano, Edilene Oliveira Silva, Vasundhra Bhandari

**Affiliations:** ^1^ Complutense University of Madrid, Madrid, Spain; ^2^ Universidade Federal do Para, Belém, Brazil; ^3^ National Institute of Pharmaceutical Education and Research, Sahibzada AjitSingh Nagar, India

**Keywords:** natural products, bacterial infections, viral infections, fungal infections, nanomedicine, protozoan infections

Infectious diseases remain among the most pressing challenges to global public health, particularly as antimicrobial resistance continues to rise and emerging pathogens threaten vulnerable populations. As traditional therapeutic approaches reach their limits, there is growing interest in next-generation strategies that merge the therapeutic potential of natural products with the precision and versatility of nanotechnology. This Research Topic brings together five diverse contributions that explore these themes from distinct yet complementary angles, ranging from innovative drug delivery systems and pharmacokinetics to meta-analyses and bibliometric insights to research trends.

A key contribution to this Topic is a comprehensive bibliometric analysis of photodynamic therapy (PDT) in anti-infective applications over the past 2 decades. Wen et al. provide the first large-scale mapping of research dynamics, identifying how the field has evolved from classical photosensitizers, such as 5-aminolevulinic acid, to cutting-edge approaches involving nanoparticles, graphene oxide, and photothermal synergy. This Review not only highlights China’s growing leadership in this area but also underscores the increasingly interdisciplinary nature of PDT-based infection control, where nanotechnology plays a vital role in enhancing therapeutic efficacy and targeting.

A second study by Suh et al. explores the antibacterial activity of bakuchiol, a plant-derived natural product, particularly against difficult-to-treat bacterial persisters. The authors demonstrate that bakuchiol effectively kills *Staphylococcus aureus* persisters and works synergistically with colistin to eliminate *Acinetobacter baumannii* persisters. Notably, bakuchiol achieves this by selectively disrupting bacterial membrane phospholipids, while sparing mammalian cells, representing a promising membrane-targeted strategy with minimal cytotoxicity. This study reinforces the potential of natural products as adjuvant therapies and highlights a novel mechanism for addressing persistent infections.

In their clinically-based contribution, Wang et al. present a population pharmacokinetic (PPK) study of imipenem in elderly Chinese patients. Given the pharmacodynamic complexity of antimicrobial therapy in aging populations, this study provides valuable data for optimizing imipenem dosing through simulations based on creatinine clearance and compartmental modeling. Though not focused directly on nanotechnology or natural products, the study fits well within the scope of the Topic by offering practical insights into precision dosing strategies, which are crucial for maximizing therapeutic outcomes and minimizing resistance.

Alsowaida et al. contribute a meta-analysis examining the effectiveness and safety of combining echinocandins with standard antifungal treatments in invasive aspergillosis. While their results did not show statistically significant improvements in clinical outcomes, the study’s findings suggest potential benefits from combination therapy, with no added safety concerns. This work provides a critical evidence-based assessment of therapeutic options for fungal infections, emphasizing the importance of rigorous clinical evaluation alongside innovation in drug design and delivery.

Finally, Queiroz-Souza et al. investigate the use of kojic acid-loaded polymeric nanoparticles (NanoFKA) against *Leishmania (Leishmania) amazonensis*, a causative agent of tegumentary leishmaniasis. Their findings demonstrate that NanoFKA induces apoptosis-like death in the parasite without affecting host macrophages. The nanoparticle formulation, using silk fibroin as a biocompatible biomaterial, exemplifies how natural product-based nanocarriers can be strategically engineered for targeted treatment of neglected tropical diseases. This approach holds promise for potentially overcoming the toxicity and complexity that are often associated with current therapies.

Together, these five articles reflect the vibrant interdisciplinary nature of next-generation therapies for infectious disease. They demonstrate how bibliometric tools, natural compound screening, advanced pharmacokinetics, meta-analytical rigor, and nanoscale delivery systems can coalesce to inform and transform clinical practice. The convergence of natural products and nanotechnology not only offers new mechanisms of action and delivery but also paves the way for more selective, potent, and safe interventions against both common and neglected infections.

We extend our sincere gratitude to the contributing authors for their high-quality work and to the Reviewers for their valuable insights throughout the process. We hope this Research Topic serves as a catalyst for further exploration and collaboration in the field of natural product-inspired nanomedicine for infectious diseases.

